# Symptomatic Thoracic Disc Herniation in a 30-Year-Old Woman

**DOI:** 10.7759/cureus.53106

**Published:** 2024-01-28

**Authors:** Isabel Fragoso, Ana Isabel Morais, Ana Isabel Almeida, Claudia A Vicente

**Affiliations:** 1 Family Medicine, Usf Araceti, Arazede, PRT; 2 Orthopaedics and Traumatology, Centro Hospitalar Universitário São João, Porto, PRT; 3 Neuroradiology, Centro Hospitalar Universitário São João, Porto, PRT; 4 Family Medicine, Grupo de Estudos de Doenças Respiratórias (GRESP), Lisbon, PRT

**Keywords:** spinal compression, paresthesia, thoracic herniation, family medicine, neurosurgery

## Abstract

Thoracic disc herniation is infrequent and presents a unique set of challenges for both diagnosis and treatment. It is an underdiagnosed entity, mainly due to the non-specific clinical manifestations. Different techniques are used for surgical treatment. This case describes a case of symptomatic thoracic disc herniation in a healthy young woman from diagnosis to surgical treatment, and it shows the importance of clinical integration and imaging studies of these cases.

## Introduction

Thoracic disc herniation, though less common than its lumbar or cervical counterparts, presents a unique set of challenges for both diagnosis and treatment. It is an underdiagnosed entity, mainly due to the non-specific clinical manifestations, with an insidious presentation, which makes diagnosis difficult and/or delayed [1,2].

About 70% of these cases are asymptomatic [1]. While a substantial proportion of these herniations may remain asymptomatic, the potential for progressive myelopathy in symptomatic instances underscores the need for intervention [3].

Thoracic hernias occur mainly at the lowest thoracic levels, between T8 and L1. Surgical treatment is indicated when the hernia occupies more than 40% of the spinal canal and there are symptoms such as back pain, intercostal neuralgia, or neurological deficits [3].

Surgical treatment is an individual strategy due to the complexity of the surgical approach to the dorsal spine and potential complications, and is highly dependent on the surgeon's experience [1,2].

This report describes a case of symptomatic thoracic disc herniation in a healthy young woman, and it aims to raise awareness of the clinical integration and imaging studies of these cases for timely referral and prevention of deficit progression.

## Case presentation

A 30-year-old Caucasian woman presented to her family physician in February 2023 complaining of non-specific discomfort in the anterior left rib cage and ipsilateral hypochondrium, exacerbated by trunk flexion, with a progression over approximately three months. The patient described the area and intensity of discomfort have increased in the last month. She had been a healthy woman and worked as a family physician. She had a cesarean section 20 months ago from a monitored low-risk pregnancy with no complications. The patient denied alcohol, smoking, and other toxicological consumption habits. She was leading a sedentary lifestyle, without regular exercise, had a body mass index of 22.6 kg/m2, and was currently using a daily combined oral contraceptive without any known drug allergies.

**Figure 1:**
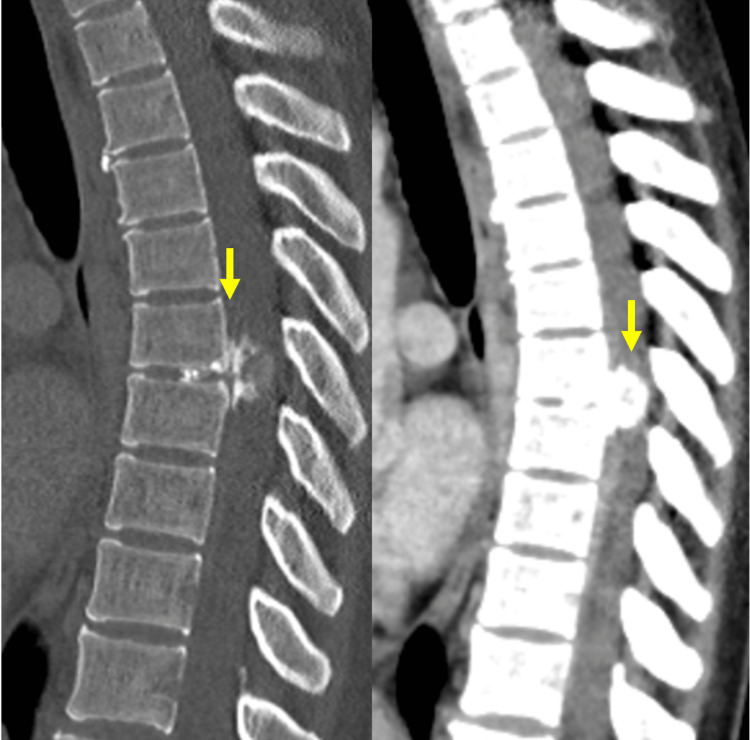
CT scan showing calcified lesion in T7 level. Arrow: calcified lesion.

On physical examination, cardio-pulmonary auscultation revealed no abnormalities and abdominal examination was unremarkable, with no identified masses or other abnormal findings. An abdominal ultrasound performed in the same month showed no pathological findings. Due to persistent and worsening symptoms, a thoracic and abdominal CT scan (March 2023) was requested and revealed a partially calcified formation at the level of T7, projecting into the medullary canal (Figure 1).

**Figure 2:**
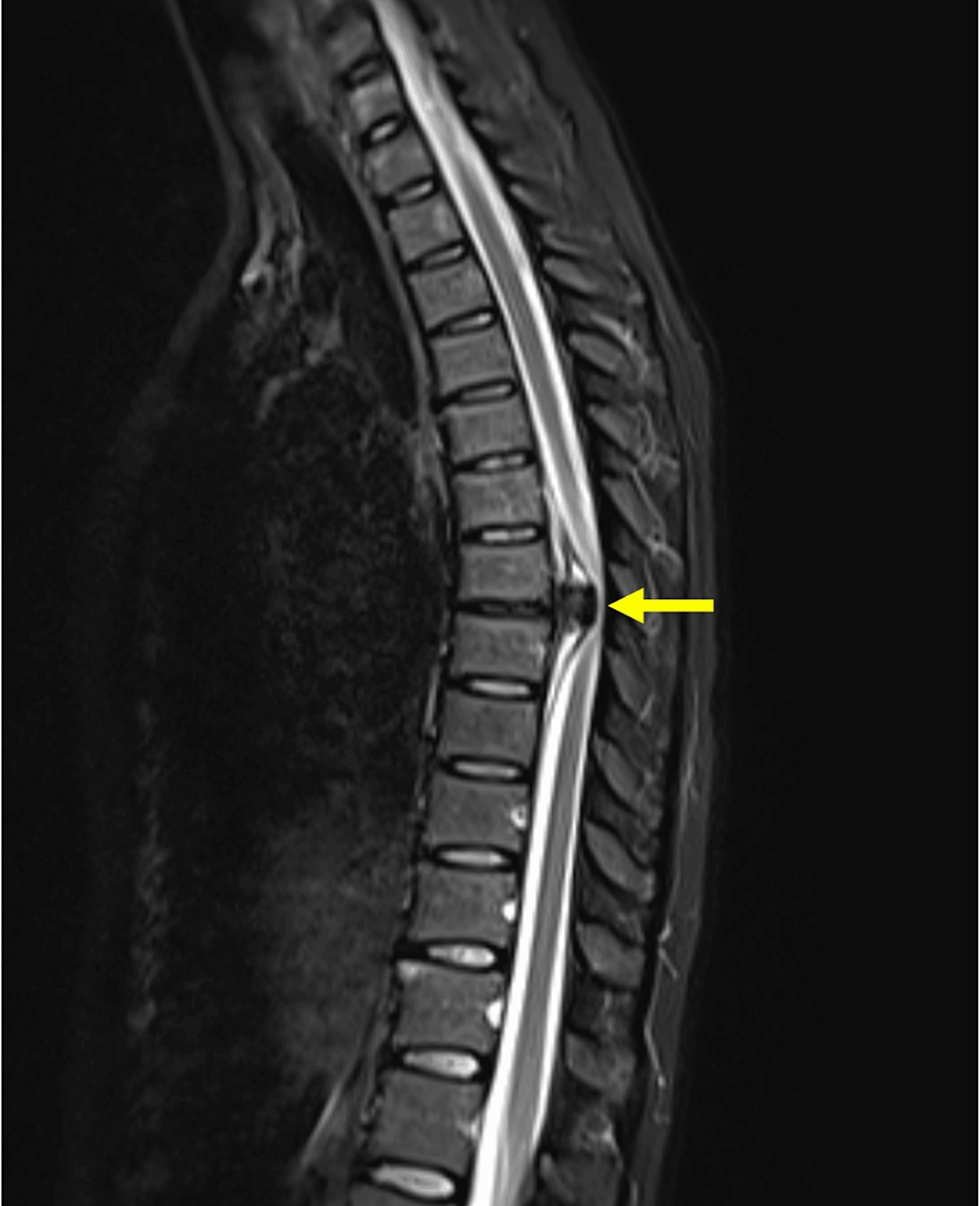
MRI sagittal image showing herniated lesion. Arrow: herniated disc.

**Figure 3:**
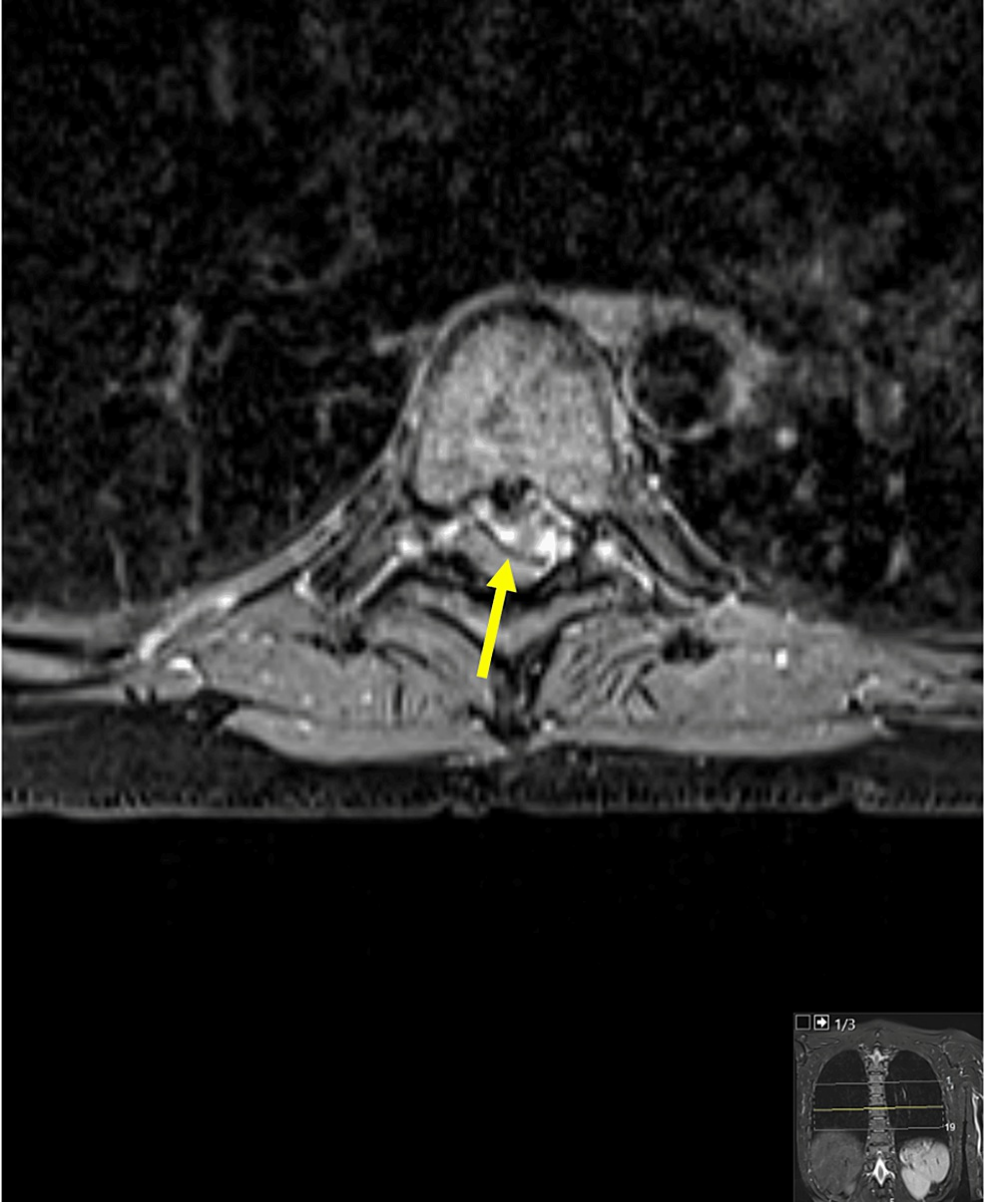
MRI transverse image (T7) showing compression of the spinal cord. Arrow: herniated lesion compressing the spinal cord.

For further characterization, an MRI of the thoracic column was performed (Figures 2, 3).

At the T6-T7 interbody space, there was a sizable extradural lesion observed, extensively calcified, as illustrated in Figure 1. This lesion exhibited well-defined edges and regular morphology. Notably, there was clear continuity in both morphology and signal with the intervertebral disc, characterized by a left paramedian posterior extrusion leading to substantial compression of the spinal cord. The leading hypothesis suggests disc extrusion as the most probable cause.

In light of these findings, the patient disclosed a history of chronic back pain over five years, previously devalued and attributed to daily activities (such as holding the baby and driving for several hours a day). Additionally, she reported transient episodes of sudden lower limb weakness described as “stumbles and failures,” which she had for several years and similarly dismissed. The neurological examination showed bilateral hyperreflexia in the lower limbs.

The patient was referred to a neurosurgery consultation, and surgery was recommended to address the herniated thoracic disc, given the degree of spinal canal compression.

**Figure 4:**
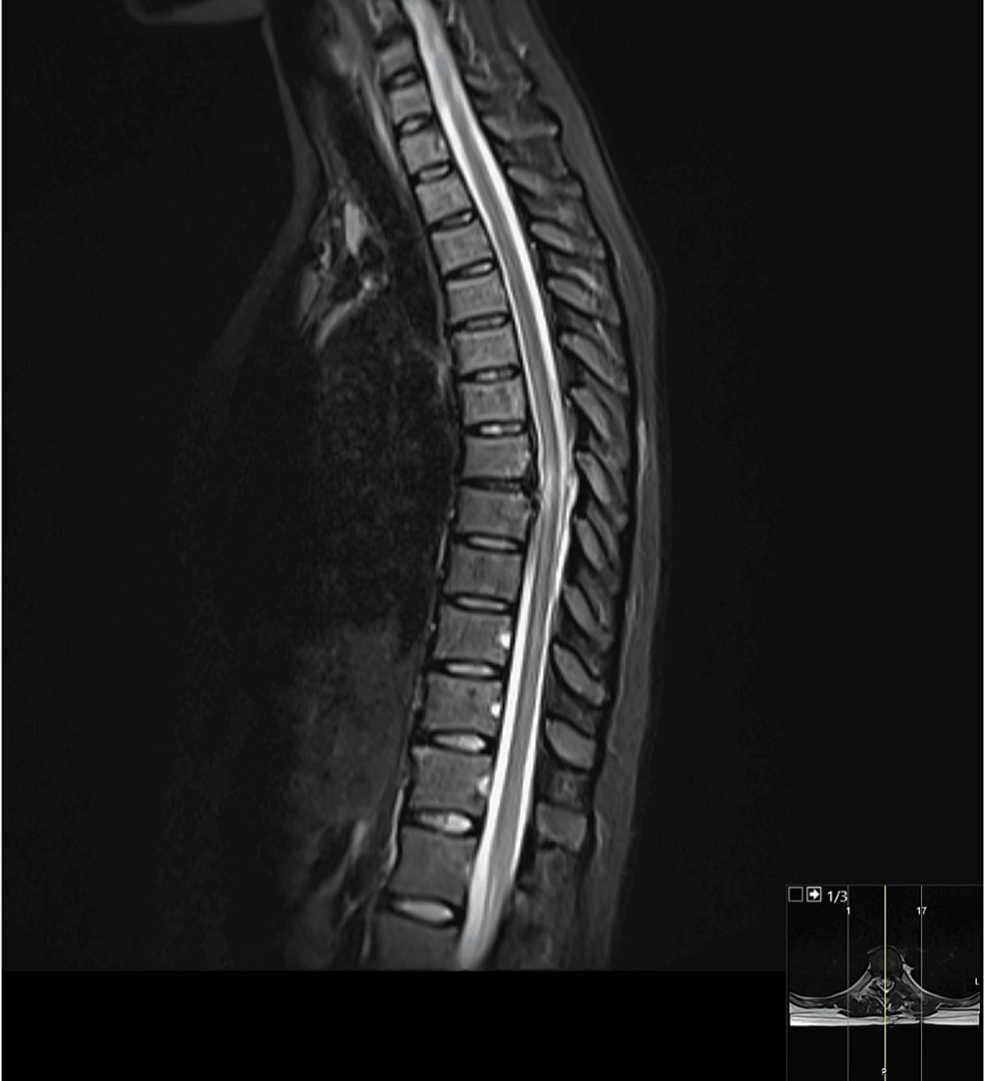
MRI two months after surgery, with no signs of recurrence.

In June 2023, the patient underwent surgery. Neurosurgeons conducted the excision of a calcified left paramedian thoracic hernia (T7-T8) utilizing a minimally invasive posterior left posterolateral approach, coupled with decompressive laminectomy at the D8 level. The postoperative period was uneventful. The patient resumed her daily activities without symptoms, including no rib cage or back pain. As anticipated, the hyperreflexia persisted. A control MRI was performed two months later (Figure 4). No signs of recurrence were detected. There were no indications of canal stenosis or spondylolisthesis. The persistent hypersignal in T2, as expected, indicates ongoing/persistent compressive myelopathy.

## Discussion

The current clinical case delineates a spinal column pathology that, while infrequent, poses a risk for myelopathy and, consequently, the development of progressive and significant neurological deficits [1].

Diagnosis is challenging, mainly due to the insidious and non-specific presentation. Due to the slow progression and atypical clinical signs, the mean period from the onset of first symptoms to diagnosis is of 15 months [4]. This makes it important to integrate the patient's complaints over time, for a correct and guided study, including requesting complementary diagnostic means, namely, imaging (CT and MRI), and subsequent referral to a specialty consultation.

Although the majority of patients respond positively to conservative treatment, it is indicated to perform surgery in some cases, including in the presence of a giant calcified hernia, which is frequently associated with the development of myelopathy [5].

In this case, because of the presence of a giant calcified hernia and neurological signs, surgery was indicated [1,4]. There are potential surgical risks, such as neurological worsening in those with previous symptoms [1], which had not occurred with this patient. Minimally invasive techniques are still growing and conquering its space in this spine level [6].

## Conclusions

While the majority of thoracic disc herniation cases may be asymptomatic, it is crucial to address symptomatic instances, to prevent progressive myelopathy. The surgical management of symptomatic thoracic disc herniation poses a significant challenge for spine surgeons due to the anatomical complexity involved and the lack of experience in some centers. There were no post-surgical sequelae, which contributes positively to the series of the described surgical approach.
